# Wafer-level meta-aspheric lenses for compact wide-FOV NIR imaging

**DOI:** 10.1038/s41377-026-02341-2

**Published:** 2026-06-02

**Authors:** Eunji Lee, Junsuk Rho

**Affiliations:** 1 Department of Chemical Engineering, Pohang University of Science and Technology (POSTECH), Pohang, 37673 Republic of Korea; 2 Department of Mechanical Engineering, Pohang University of Science and Technology (POSTECH), Pohang, 37673 Republic of Korea; 3 Department of Electrical Engineering, Pohang University of Science and Technology (POSTECH), Pohang, 37673 Republic of Korea; 4 POSCO-POSTECH-RIST Convergence Research Center for Flat Optics and Metaphotonics, Pohang, 37673 Republic of Korea

**Keywords:** Metamaterials, Nanophotonics and plasmonics

## Abstract

A meta-aspheric lens fabricated at the wafer-level achieves a 101.5° field of view, a 3.39 mm total track length, and an F/1.64 aperture within a volume of 0.02 cm^3^, enabling compact and scalable near-infrared imaging.

## Abstract (web summary)

A wafer-level meta-aspheric lens integrates refractive and meta-optical elements to realize ultracompact, wide-field near-infrared imaging for next-generation vision modules.

## Graphical abstract

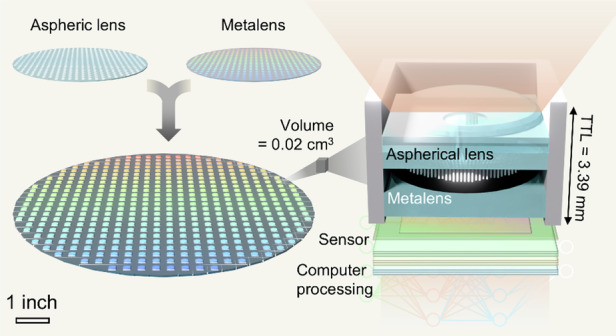

Near-infrared (NIR) imaging is now integral to consumer and biomedical systems, spanning AR eye tracking, biometric authentication, and subcutaneous vascular imaging^1–4^. As these platforms demand thinner and lighter form factors, conventional refractive optics face a fundamental limitation. Even the most advanced refractive optics require additional elements to correct optical aberrations, inevitably increasing system volume and assembly complexity^5^.

Metalenses are ultrathin flat optical elements comprising subwavelength nanostructure arrays. Owing to their ability to precisely control the phase, amplitude, and polarization of light within a planar geometry, they have emerged as a promising platform for miniaturized imaging systems^6–9^. However, single-layer metalenses face inherent trade-offs between aperture size, field of view, and aberration correction^10,11^.

One way to overcome this is to combine multiple flat elements, distributing the burden across them. In 2016, Arbabi et al. demonstrated that combining two metasurfaces into a doublet could simultaneously correct wide-angle monochromatic aberrations, establishing a foundational principle for multi-element meta-optical design^12^. Building on this, in 2018, Chen et al. combined a spherical lens with a metasurface corrector to extend achromatic performance into the NIR^13^. Balli et al. demonstrated a hybrid achromatic metalens with improved focusing efficiency^14^. Sawant et al. extended this to centimeter-scale hybrid metalenses, simultaneously correcting chromatic and spherical aberrations^15^.

To date, no single integrated platform has simultaneously achieved a FOV exceeding 100°, a TTL below 5 mm, and wafer-level manufacturability. This combination is critical for next-generation compact NIR imaging modules. Writing in *Light: Advanced Manufacturing*, Chi et al. report a meta-aspheric lens (MAL) that achieves all three. An aspherical refractive lens and a metalens are monolithically integrated and bonded at the wafer level^16^, requiring only one dicing step and no post-fabrication mechanical alignment.

As illustrated in Fig. 1 (left), the aspherical refractive lens is produced via laser direct writing and nanoimprint lithography on a separate wafer. Then aligned and bonded to the metalens at the micrometer level. An engineered air gap above the metasurface protects the nanostructures during bonding. A forward model that incorporates the experimentally measured dispersion of the $\alpha$-Si material ensures close agreement between simulation and fabrication. The resulting MAL imaging system (Fig. 1, right) achieves a 101.5° FOV, a 3.39 mm TTL, and an F/1.64 aperture within a total volume of just 0.02 cm^3^, maintaining an MTF exceeding 0.31 at 50 lp/mm across the entire field. Imaging demonstrations span USAF resolution targets, eye-model tracking across multiple gaze angles, and visualization of dorsal hand veins invisible to conventional visible-light cameras. Additionally, the authors demonstrate computational super-resolution using the MambaIR deep learning model, which they report as the first application to NIR imaging^17^.

**Fig. 1:**
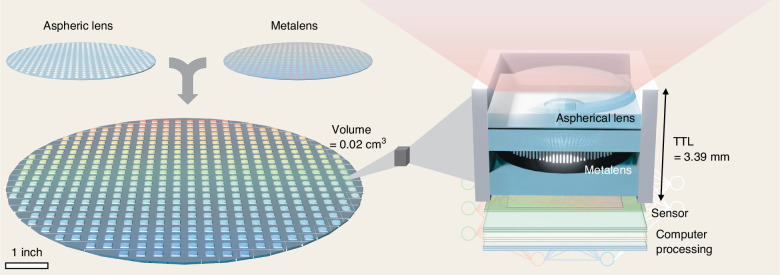
Wafer-level fabricated meta-aspheric lens (MAL) and imaging system. (Left) Schematic of the wafer-level bonding, in which an aspheric lens wafer and a metalens wafer are monolithically integrated into a single MAL wafer, enabling wafer-level mass fabrication. (Right) Cross-sectional schematic of the MAL imaging system, consisting of an aspherical lens, aperture, metalens, air gap, cover glass, and image sensor within a field-of-view (FOV) of 101.5°, total track length (TTL) of 3.39 mm, and a total volume of 0.02 cm^3^

Transitioning from electron beam lithography to higher-throughput patterning approaches such as deep-UV lithography or nanoimprint-based metalens fabrication will be an important next step for further reducing cost and increasing production yield^18–20^. Extending the operational bandwidth beyond the current range will broaden applicability to multispectral NIR tasks, including blood oxygen imaging and night vision. This platform could be adapted for facial recognition, iris scanning, and gaze estimation modules in next-generation AR glasses, where an ultracompact form factor and a wide FOV are simultaneously required.
